# Decreased Regulatory T Cells in Vulnerable Atherosclerotic Lesions: Imbalance between Pro- and Anti-Inflammatory Cells in Atherosclerosis

**DOI:** 10.1155/2015/364710

**Published:** 2015-01-15

**Authors:** Ilonka Rohm, Yevgeniya Atiskova, Stefanie Drobnik, Michael Fritzenwanger, Daniel Kretzschmar, Rudin Pistulli, Jürgen Zanow, Thomas Krönert, Gita Mall, Hans Reiner Figulla, Atilla Yilmaz

**Affiliations:** ^1^Department of Internal Medicine I, Friedrich-Schiller-University, 07747 Jena, Germany; ^2^Institute of Forensic Medicine, Friedrich-Schiller-University, 07743 Jena, Germany; ^3^Department of Vascular Surgery, Friedrich-Schiller-University, 07747 Jena, Germany; ^4^Department of Vascular Surgery, Thüringen Klinik Saalfeld, 07318 Saalfeld, Germany

## Abstract

Atherosclerosis is a chronic inflammatory disease of the arterial wall in which presentation of autoantigens by dendritic cells (DCs) leads to the activation of T cells. Anti-inflammatory cells like Tregs counterbalance inflammation in atherogenesis. In our study, human carotid plaque specimens were classified as stable (14) and unstable (15) according to established morphological criteria. Vessel specimens (*n* = 12) without any signs of atherosclerosis were used as controls. Immunohistochemical staining was performed to detect different types of DCs (S100, fascin, CD83, CD209, CD304, and CD123), proinflammatory T cells (CD3, CD4, CD8, and CD161), and anti-inflammatory Tregs (FoxP3). The following results were observed: in unstable lesions, significantly higher numbers of proinflammatory cells like DCs, T helper cells, cytotoxic T cells, and natural killer cells were detected compared to stable plaques. Additionally, there was a significantly higher expression of HLA-DR and more T cell activation (CD25, CD69) in unstable lesions. On the contrary, unstable lesions contained significantly lower numbers of Tregs. Furthermore, a significant inverse correlation between myeloid DCs and Tregs was shown. These data suggest an increased inflammatory state in vulnerable plaques resulting from an imbalance of the frequency of local pro- and anti-inflammatory immune cells.

## 1. Introduction

Atherosclerosis can be defined as an inflammatory process: the exposure of extracellular matrix proteoglycans facilitates the subendothelial accumulation of low-density lipoprotein (LDL) which is then exposed to oxidation. Oxidized LDL (oxLDL) activates endothelial cells and stimulates them to secrete chemokines and express adhesion molecules which lead to the extravasation of different immune cells, such as monocytes and T cells. After migration into the intima, monocytes differentiate into macrophages which take up oxidized lipids and thereby transform into foam cells. The growing accumulation of cell debris and lipids leads to the formation of a necrotic plaque core. Smooth muscle cells migrate from the media into the intima where they produce extracellular matrix (ECM) proteins which compose a fibrous cap covering the plaque core, thereby stabilizing the atheroma (plaque stabilization). Macrophages lead to the thinning of this fibrous cap through the release of matrix metalloproteinases which is the prerequisite of plaque rupture (plaque destabilization). Plaque rupture is followed by acute ischemic events such as stroke or acute myocardial infarction. However, the reason for inflammation in atherosclerosis was unknown for a long time. In recent times, it has been unraveled that certain autoantigens like oxLDL might be the trigger for chronic inflammation in atherosclerosis. Autoantigens are presented in atherosclerotic lesions by antigen-presenting cells (APC) like macrophages or DCs and recognized by T cells. T cells in turn contribute to the inflammatory state through the secretion of different proinflammatory mediators [1].

It has been shown that DCs as professional APCs are present in atherosclerotic lesions and that they are essential for the initiation of an autoimmune process through activation of T cells. There are two major subpopulations of DCs: myeloid DCs (mDCs) and plasmacytoid DCs (pDCs). mDCs mainly recognize bacterial fragments and oxidized autoantigens, and pDCs are specialized in sensing viral fragments. Autoantigens like oxLDL promote the maturation of DCs, enabling them to trigger an antigen-specific T cell activation. IFN-*α*, secreted by pDCs, correlates with plaque instability and stimulates naïve CD4+ T cells to differentiate into cytotoxic T cells and express IFN-*γ*, a potent regulator of T cell function [2]. In bioengineered arteries, activated mDCs stimulate autologous CD4+ T cells to produce IFN-*γ*, infiltrate the vessel wall, and cause inflammation [3]. However, it has been shown recently that DCs are also able to induce antigen-specific tolerance in peripheral T cells, which is necessary to suppress the progression of atherosclerosis [4, 5].

Within the T cell subset, the majority of pathogenic cells in atherosclerotic lesions belong to the T helper (Th) 1 profile, producing proinflammatory mediators such as IFN-*γ*. The role of Th2 cells in atherogenesis remains controversial. There is now accumulating evidence that within the T cell population there is also a subset of specialized T cells with anti-inflammatory properties: regulatory T cells (Tregs). Tregs are known to play a critical role in the control of inflammation and autoimmunity including chronic vascular inflammation causing atherosclerosis. An increase in Tregs was shown to correlate with a reduction in atherosclerosis in animal models [6], and Tregs depletion promoted atherosclerosis in mouse models [7]. Naturally occurring Tregs produce IL-10 and TGF-*β*. These cytokines were shown to be protective regarding plaque development [8].

A theory gaining more and more acceptance trying to explain the reason for atherogenesis is the imbalance between immune cells producing proatherogenic mediators and regulatory T cells with immunosuppressive, anti-atherogenic properties.

The aim of our study was to answer the following questions: (1) whether the presence of certain immune cells is associated with the presence of other pro- or anti-inflammatory cells, for example, myeloid or plasmacytoid DCs with proinflammatory T cells or anti-inflammatory Tregs, and (2) whether the expression of functional molecules such as HLA-DR, CD25, and different chemokine receptors which might be involved in the attraction of circulating immune cells correlates with the plaque stability.

## 2. Methods

### 2.1. Patients

Plaque specimens of 29 patients undergoing endarterectomy of elastic arteries were analyzed in our present study. Indications for carotid endarterectomy (*n* = 17) were based on NASCET and ACAS criteria [9, 10]. Indications for femoral endarterectomies (*n* = 12) were high grade stenoses < 3–10 cm length causing relevant reductions in the walking distance [11]. Duplex scanning, magnetic resonance imaging, or angiography was performed to quantify the degree of stenosis prior to surgery. The study was approved by the local ethics committee and conducted in concordance with the Declaration of Helsinki. All patients gave informed written consent. Clinical data are listed in Table 1.

As controls, 12 vessel specimens of elastic arteries were obtained from accident or suicide victims who did not show any macroscopic or histological signs of atherosclerosis.

### 2.2. Histological Analysis

**Figure 1:**
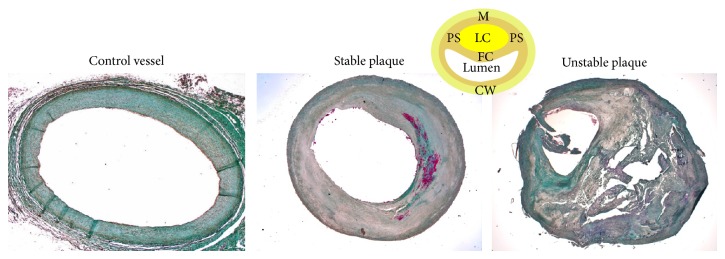
Trichrome staining of atherosclerotic plaques enabling histological classification in stable or unstable lesions. Trichrome staining (20x) of a control vessel, a stable plaque with mainly fibrous tissue and a thick fibrous cap, and an unstable plaque with a large lipid core and/or a thin fibrous cap. Scheme of an atherosclerotic plaque demonstrating the different plaque regions is also shown: lipid core (LC), plaque shoulders (PS), fibrous cap (FC), media (M), and contralateral wall (CW).

Endarterectomy specimens were fixed in 4% formalin. Areas with extensive plaque formation were cut out for further analyses. Sections with total vessel occlusion were excluded from the study. Plaques used in this study were at an advanced stage (types IV to VI) according to the AHA classification [12]. After decalcification in EDTA for 4 weeks, plaques were paraffin-embedded. Serial sections (4 *μ*m) were cut and mounted on glass slides. Trichrome staining was performed to analyze plaque morphology and plaque regions as previously described [13] (Figure 1). The fibrous cap was defined as the area between the lipid core and the lumen. The lipid core was defined as the inner, in unstable plaques lipid-rich and necrotic part. The plaque shoulders were located where the fibrous cap hits the regular vessel wall at an angle of approximately 90°. Media was considered the fibrous area surrounding the lipid core. All specimens were analyzed blinded to the clinical symptoms and the identity of each patient. The sizes of the lipid core area (mm^2^) and of the total plaque area (mm^2^) were measured by computer aided planimetry (Image J 1.43u, Wayne Rasband, NIH, USA). The lipid core ratio (LCR, %) was calculated (lipid core area/plaque area × 100). The fibrous cap was measured at its narrowest site. According to histological criteria [14], plaque specimens were histologically classified as stable, fibrous lesions (fibrous cap > 100 *μ*m, LCR < 40%, ≤ 3 neovessels/0.2 mm^2^), or unstable, vulnerable, lipid-rich plaques (fibrous cap < 100 *μ*m, LCR > 40%, neovascularization, >3 neovessels/0.2 mm^2^) (Table 2).

### 2.3. Immunohistochemical Stainings

The antibodies used for immunohistochemical staining are listed in Table 3, and catalyzed signal amplification technique (CSA System, DakoCytomation, Hamburg, Germany) was used according to manufacturer's instructions. CD34 immunostaining helped to detect neovascularization as a criterion for plaque instability [15]. The sections were treated with irrelevant isotype-matched antibodies as appropriate negative controls.

### 2.4. Quantification of Immunostained Cells

Digital images of different plaque regions (magnification 200x) were taken with a CCD-camera (Zeiss AxioCam HRC, Jena, Germany). Cells were digitally counted in defined random areas (0.1 mm^2^) in each plaque region using a digital image processing software (Axiovision, Zeiss, Jena, Germany). For each quantification, the color threshold for immunostained cells was manually adjusted until the computerized detection matched the visual interpretation. The mean cell number per plaque was calculated from the cell numbers assessed in the different plaque regions. The results showed an intra- and interobserver variability of less than 10%.

### 2.5. Statistical Analysis

Statistical analysis was performed with SigmaPlot Software Version 12.0 (Systat Software Inc.). All values are reported as median; *P* < 0.05 was considered statistically significant. The nonparametric Mann-Whitney Rank Sum Test was used to compare the number of different cells between the different study groups. Correlation analyses were performed using Spearman Rank Order Test.

## 3. Results

In this study, the cellular composition of 29 advanced plaques that were classified as stable or unstable according to established criteria was immunohistochemically analyzed. After immunohistochemical staining, the frequencies of different immune cells as well as functional markers were compared between unstable plaques (*n* = 15), stable plaques (*n* = 14), and vessels without any signs of atherosclerosis that served as control (*n* = 12). For absolute cell numbers and *P* values see Table 4.

### 3.1. Emergence of DCs in Stable and Unstable Atherosclerotic Lesions

**Figure 2:**
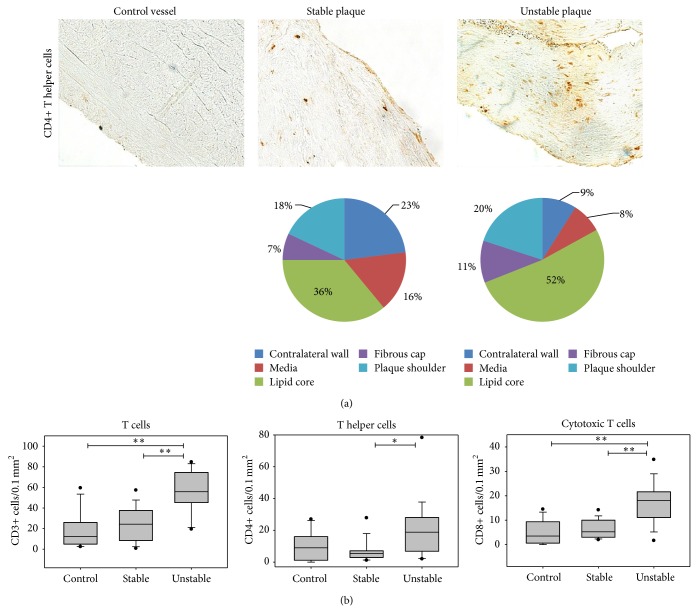
Emergence of myeloid DCs in atherosclerotic plaques. (a) Immunohistochemical staining of fascin+ mDCs of a control vessel and of the plaque shoulder regions of a stable and an unstable plaque (200x). The pie diagrams demonstrate the percentage of immunostained cells present in different plaque regions (LC—lipid core, PS—plaque shoulders, FC—fibrous cap, M—media, and CW—contralateral wall). (b) Mean DC number of control vessels (*n* = 12), stable (*n* = 14), and unstable (*n* = 15) plaques. Results are expressed as cells per 0.1 mm^2^. Values are presented as median (25–75% CI), ^*^ *P* < 0.05, ^**^ *P* < 0.01 (mDCs—myeloid DCs, pDCs—plasmacytoid DCs).

Macrophages (CD68+) as typical and well-known APCs were significantly more often present in advanced atherosclerotic lesions than in healthy vessels (Table 4). To investigate the frequency of dendritic cells (DCs) as other important APCs in atherosclerosis, immunostaining of plaques with different DC markers was performed (Table 4, Figure 2). To investigate the global emergence of myeloid (m) DCs, the expression of fascin and S100 was analyzed. The number of fascin+ mDCs was significantly higher in unstable than in stable plaques (1.6-fold) or control vessels (1.9-fold). The frequency of S100+ DCs was also significantly 1.6-fold higher in unstable than in stable plaques. This supports the theory of an increase in the number of mDCs in the course of plaque destabilization. To further investigate the maturation level of DCs during the process of plaque destabilization, immunohistochemical staining was performed with markers that allow the differentiation between mature mDCs (CD83) and immature mDCs (CD209). For mature mDCs, a higher emergence was visible in stable plaques compared to control vessels (1.6-fold), and a further significant increase was visible in emergence in unstable compared to stable lesions (5.9-fold). The significant increases in the cell number of immature mDCs in the course of plaque progression were lower than those of mature mDCs (Table 4). On the contrary, for pDCs (CD123+, CD204+), no significant difference was detected between unstable and stable plaques.

In addition to the immunostaining with cellular markers, we also performed immunostaining with HLA-DR, a functional APC marker which is upregulated through activation. The frequency of HLA-DR expressing cells was significantly higher in unstable than in stable plaques (1.6-fold) and control vessels (2.4-fold).

### 3.2. Frequency of T Cells in Different Stages of Atherosclerotic Lesions

#### 3.2.1. Proinflammatory T Cells

**Figure 3:**
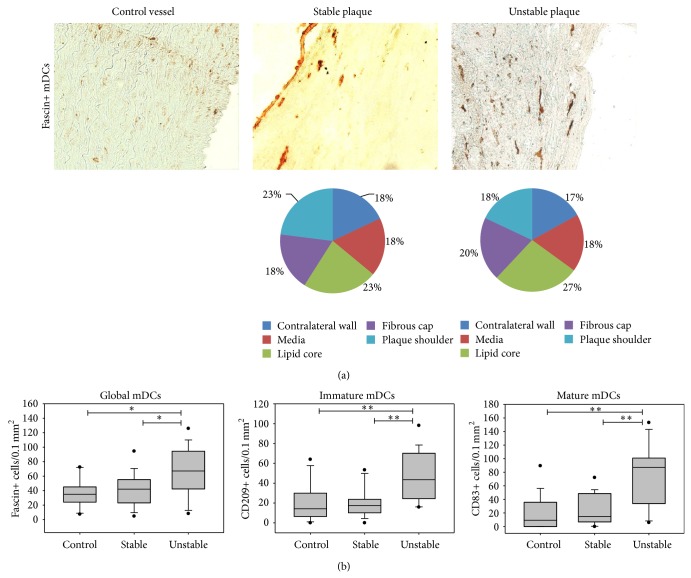
T cell emergence in atherosclerotic lesions. (a) Immunohistochemical staining of proinflammatory CD4+ T helper cells of a control vessel and of the plaque shoulder regions of a stable and an unstable atherosclerotic plaque (200x). The pie diagrams demonstrate the percentage of immunostained cells present in each plaque region (LC—lipid core, PS—plaque shoulders, FC—fibrous cap, M—media, and CW—contralateral wall). (b) Mean T cell numbers of control vessels (*n* = 12), stable (*n* = 14), and unstable (*n* = 15) plaques. Results are expressed as cells per 0.1 mm^2^. Values are presented as median (25–75% CI). ^*^ *P* < 0.05, ^**^ *P* < 0.01.

The frequency of T cell subgroups was also compared for stable and unstable atherosclerotic plaques (Table 4, Figure 3). The number of CD3+ T cells was significantly higher in unstable than in stable lesions (2.3-fold) or control vessels (4.5-fold). Also, CD4+ T helper cells and CD8+cytotoxic T cells occurred significantly more often in unstable than in stable lesions (3.4-fold). Furthermore, the emergence of natural killer T cells as another T cell subset playing an important role in atherosclerosis was investigated by immunostaining with CD161. A significantly higher emergence of these cells in unstable than stable lesions (1.5-fold) or healthy vessels (6.3-fold) was observed.

#### 3.2.2. Activated T Cells

CD25 is expressed by activated B and T cells, including Tregs. The CD25 expression was investigated in advanced lesions comparing unstable and stable plaques. The number of CD25+ T cells was significantly 6.6-fold higher in vulnerable than fibrous atherosclerotic lesions. As an early activation marker which is also expressed by activated B and T cells as well as macrophages and platelets, occurrence of CD69 was investigated. There was a significant 1.7-fold higher number of CD69+ cells in unstable than stable plaques (Table 4).

#### 3.2.3. Anti-Inflammatory T Cells

**Figure 4:**
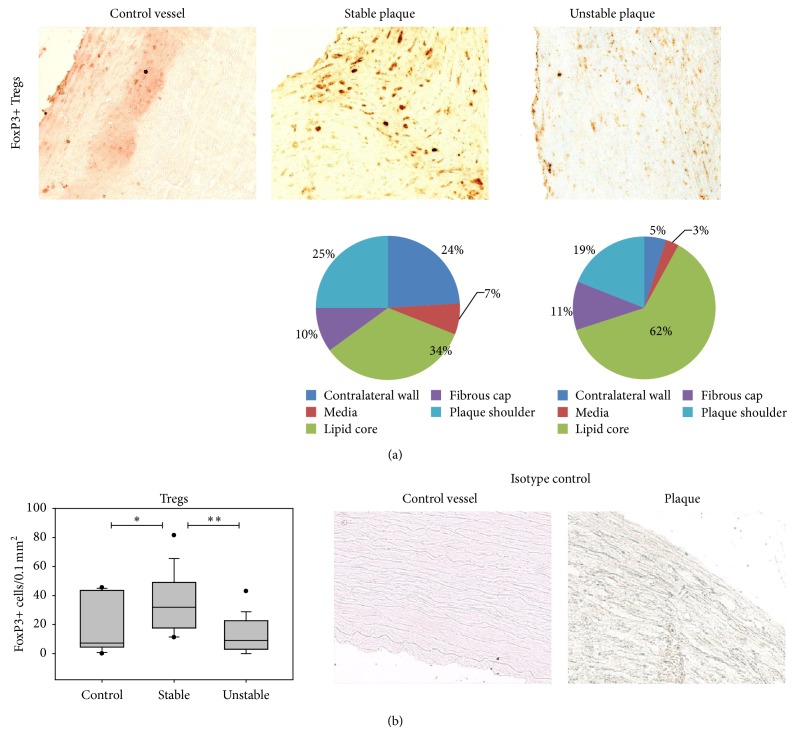
Emergence of Tregs in atherosclerotic lesions. (a) Immunohistochemical staining of Tregs of a control vessel without atherosclerosis and the plaque shoulder regions of a stable and an unstable atherosclerotic lesion (200x). The pie diagrams demonstrate the percentage of immunostained cells present in each plaque region (LC—lipid core, PS—plaque shoulders, FC—fibrous cap, M—media, and CW—contralateral wall). (b) Mean Treg number of control vessels (*n* = 12), stable (*n* = 14), and unstable (*n* = 15) plaques. Results are expressed as cells per 0.1 mm^2^. Values are presented as median (25–75% CI), ^*^ *P* < 0.05, ^**^ *P* < 0.01 (Tregs—regulatory T cells). Right lower corner: isotype control of a control vessel and a plaque.

In contrast to the observations of higher cell numbers of DCs and proinflammatory T cells, the number of FoxP3+ Tregs was significantly lower in vulnerable than in stable plaques (3.5-fold decrease) (Table 4, Figure 4).

### 3.3. Expression of Chemokine Receptors in Advanced Plaques

In addition to certain surface molecules of different immune cells, the expression of different chemokine receptors was investigated to analyze the role of chemoattraction during atherogenesis. CCR6 is expressed on immature DCs. A significantly higher 2.1-fold number of CCR6+ cells was observed in unstable compared to stable lesions or control vessels. CCR4 is expressed on DCs and T and B cells. The emergence of CCR4+ cells was significantly higher in vulnerable than fibrous lesions (2.1-fold) or control vessels (2.7-fold). This demonstrates a potential role of chemokine receptors in the recruitment of proinflammatory cells aggravating vascular inflammation.

### 3.4. Correlation Analyses of Proinflammatory and Anti-Inflammatory Cells

Several significant correlations between different proinflammatory cells were observed in advanced plaques, for example, correlations between different subtypes of T cells and subsets of DCs (Table 5). However, there were also many significant correlations between T cell subsets and mDCs, for example, fascin+DCs-CD4+ cells, fascin+DCs-CD161+ cells, CD209+DCs-CD8+ cells, and CD83+DCs-CD4 cells (correlation coefficients [*r*-values] > 0.6, *P* values < 0.001). This shows that several proinflammatory cells are equally attracted into the vessel wall during atherogenesis. Also, the functional marker of APC HLA-DR correlates with the marker of activation for T cells CD25 (*r* = 0.67, *P* < 0.001), implicating that not only the attraction of these cells but also their activation significantly correlates in atherogenesis.

To investigate chemoattraction, the correlation between proinflammatory cells and chemokine receptors was investigated. There were significant correlations found for proinflammatory cells and chemokine receptors: CCR4+cells-CD3+cells (*r* = 0.56, *P* = 0.002), CCR4+cells-CD25+cells (*r* = 0.48, *P* = 0.01), CCR4+cells-CD4+cells (*r* = 0.45, *P* = 0.02), and CCR6+cells-CD8+cells (*r* = 0.46, *P* = 0.01). These positive correlations between chemokine receptors and different proinflammatory cells during plaque destabilization implicate a possible role of chemoattraction of these cells into the atherosclerotic plaque.

**Figure 5:**
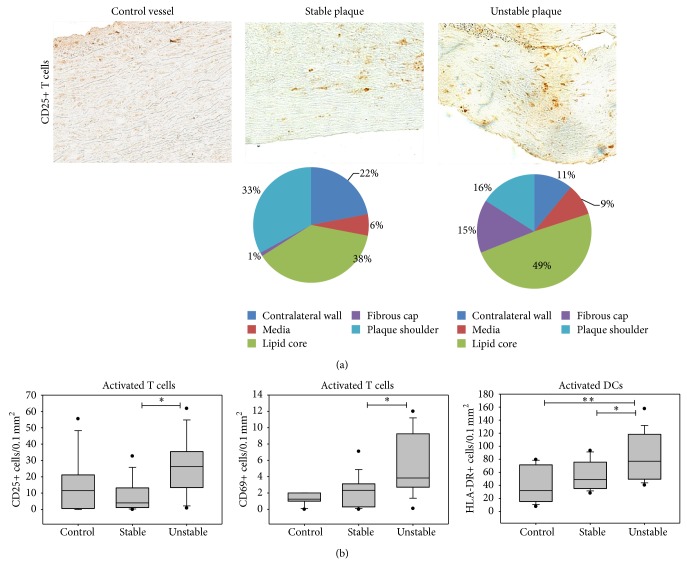
Emergence of activated cells in atherosclerotic lesions. (a) Immunohistochemical staining of activated CD25+ T cells of a control vessel without atherosclerosis and of the plaque shoulder regions of a stable and an unstable atherosclerotic lesion (200x). The pie diagrams demonstrate the percentage of immunostained cells present in each plaque region (LC—lipid core, PS—plaque shoulders, FC—fibrous cap, M—media, and CW—contralateral wall). (b) Mean CD25+ activated T cell, mean CD69+ activated T cell, and mean HLA-DR+ DC number of control vessels (*n* = 12), stable (*n* = 14), and unstable (*n* = 15) plaques. Results are expressed as cells per 0.1 mm^2^. Values are presented as median (25–75% CI), ^*^ *P* < 0.05, ^**^ *P* < 0.01.

Contrary to the positive correlations between different subsets of proinflammatory cells, a significant inverse correlation was visible between anti-inflammatory FoxP3+ Tregs and proinflammatory cells such as CD4+ T-helper cells (*r* = −0.40, *P* = 0.03), CD83+ (*r* = −0.39, *P* = 0.04), and CD209+ (*r* = −0.43, *P* = 0.04) DCs as well as chemokine receptors such as CCR4 (*r* = −0.38, *P* < 0.05) (Figure 5).

### 3.5. Correlation of the Emergence of Different Immune Cells with Clinical Data

**Figure 6:**
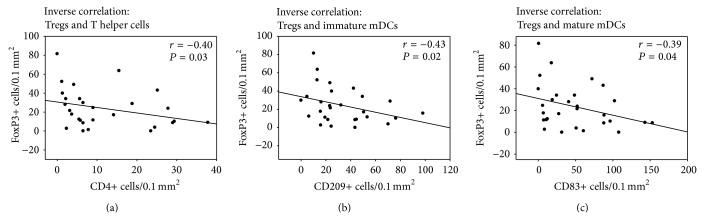
Inverse correlation between the median numbers of Tregs and proinflammatory cells in atherosclerotic plaques. The correlation analyses show the significant inverse correlation between the median number of Tregs and T helper cells (a), mature mDCs (b), and immature mDCs (c).

**Figure 7:**
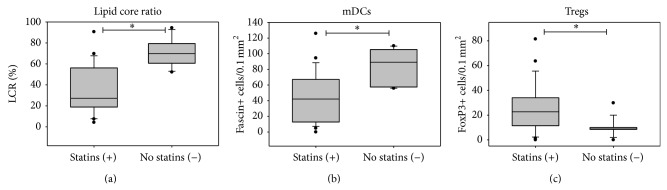
Plaque stability and preoperative statin treatment. (a) Median lipid core ratio (LCR = lipid core area/plaque area × 100, (a)) as a plaque stability criteria, median number of fascin+ DCs/0.1 mm² (b), and FoxP3+ Tregs/0.1 mm² (c) of/in plaques of patients with (+) and without (−) preoperative statin medication.

There was no significant correlation between the frequency of the cells investigated and cardiovascular risk factors or ischemic symptoms (data not shown). Interestingly, a preexisting statin therapy might influence the frequency of immune cells in plaques. Statin-treated patients showed a trend to a more stable plaque morphology, a significantly decreased number of fascin+ and CD83+ DCs (2.1-fold decrease, *P* = 0.006; 3.6-fold decrease, *P* = 0.04), and a significantly higher emergence of FoxP3+ Tregs (2.6-fold increase, *P* = 0.04) (Figure 6). This decrease in proinflammatory cells and increase in cells with anti-inflammatory properties underline the plaque-stabilizing effects of statins (Figure 7).

### 3.6. Emergence of Certain Immune Cells in Different Regions of the Plaque

For T helper cells, plaque destabilization is attended by an increase of cells in the lipid core and the plaque shoulders as well as a reduction in the tunica media (Figure 3). This can be explained by the fact that oxidized lipid present in the lipid core is one of the major triggers attracting T cells into the vascular wall. As shown in Figure 2, the distribution of fascin+ DCs is almost equal in plaques with stable and unstable morphology. In unstable plaques, Tregs seem to be mainly in the lipid core. In rupture-prone shoulders there is a lower relative and absolute number of Tregs present in unstable lesions (Figure 4).

## 4. Discussion

Atherosclerosis as an inflammatory disease of the vessel wall involves different types of immune cells. In a former study, Yilmaz et al. [13] showed that DCs as very potent APCs play an important role during plaque destabilization. There is also accumulating evidence that Tregs as anti-inflammatory, tolerance-inducing T cells are involved in atherogenesis [16]. Recently, DCs were shown to be able to induce antigen-specific tolerance in peripheral T cells [4, 5, 17]. This raises the question in which way DCs and T cells interact during atherogenesis, as inductor of pro- or anti-inflammatory T cells.

Therefore, the aim of the present study was to analyze the frequency of different immune cells in advanced plaques subdivided in stable und unstable lesions according to histological criteria [13] and investigate whether the presence of DCs is associated with the presence of pro or anti-inflammatory cells. Furthermore the expression of functional molecules and different chemokine receptors were analyzed to get insights in possible mechanisms of attraction. The following results were observed.

As expected, in unstable plaques we detected significantly higher numbers of mDCs, T cells, T helper cells, cytotoxic T cells, natural killer cells, and activated T cells compared to stable lesions. This shows that plaque destabilization is accompanied by an increase of proinflammatory cells as shown in former studies [18]. A recent study showed a coaccumulation of DCs and natural killer cells during plaque progression [19]. Within mDCs, mature DCs are able to cause an immune response through antigen-specific T cell activation, and immature DCs are thought to mediate tolerance [20, 21]. We were able to show a higher number of mature mDCs in unstable compared to stable atherosclerotic lesions which show an increase in vascular inflammation in the course of plaque progression. The equal number in the frequency of pDCs in stable and unstable plaques is not surprising. Former studies showed that pDCs are not recruited into inflammatory but rather lymphatic tissue [22, 23]. pDCs were formerly described to be present in atherosclerotic plaques and suggested to activate T cells [2]. However, apparently they do not seem to play a major role in atherogenesis [24].

Additionally, the emergency of Tregs as important anti-inflammatory cells was analyzed. There was a low number of Tregs present in control vessels and a significant 4.4-fold increase in Tregs frequency in stable plaques was obvious. An explanation might be that in healthy vessels only few inflammatory cells are present. Atherogenesis then goes along with recruitment of immune cells to the lesion site [25]. Then in the course of plaque destabilization a decrease in Treg frequency to less than a third was evident. This inverse correlation between Tregs and plaque rupture suggests an anti-inflammatory function of these cells in atherosclerosis.

Tregs can be divided into two entities: thymus-derived natural Tregs and peripherally generated induced Tregs. In our study, Tregs have been characterized by the marker FoxP3 which is expressed by both subgroups and thus does not allow us to differentiate between natural and induced Tregs. Recently, novel markers have been found that might help to characterize natural Tregs: natural Tregs have a higher expression of neuropilin 1 and Helios compared to induced Tregs [26]. In further experiments, these new markers should be investigated in IHC staining experiments to further analyze Tregs in atherosclerotic plaques and their role in atherogenesis.

So far, to our knowledge only three papers investigating the number of FoxP3+ Tregs have been published [27–29]. de Boer et al. [27] compared advanced lesions to early lesions and observed significantly increased numbers of Tregs in vulnerable, high risk compared to early lesions. There was no significant difference between vulnerable and fibrosclerotic lesions found. Patel et al. [28] observed higher numbers of Tregs in plaques from patients with ischemic symptoms than asymptomatic patients. Because of similar clinical data of the included patients, protocol differences of cell analyses might be a reason for the discrepancy of the results of these studies and our results. In the present study, five plaque regions were separately evaluated for each plaque, whereas de Boer et al. evaluated the emergence of Tregs only in the intima or adventitia. Patel et al. analyzed twenty fields per section, but it is not specifically mentioned which plaque regions the fields originated from. Recently, a study was published which revealed very similar results compared to our own investigations. Dietel et al. [29] showed decreased numbers of Tregs in unstable plaques and an inverse correlation with mDCs. For immunostaining, Dietel et al. used other DC markers (CD11c and DC-LAMP) compared to our study, showing that the results are independent of a special marker used. In contrast to Dietel et al., in the present study different DC subsets were analyzed. We characterized not only myeloid, but also plasmacytoid DCs (CD123+, CD304+) and different stages of maturation. Furthermore, different T cell subsets were investigated in our study: T helper cells, cytotoxic T cells, natural killer cells, and CD25+ as well as CD69+ activated T cells.

Immunostaining with antibodies against different chemokine receptors revealed a correlation between CCR6+ and CD8 cells. Also, a correlation between CCR4+ and proinflammatory T cells and mDCs and an inverse correlation between CCR4+ cells and Tregs were visible. This might support the idea that these CCRs have an important function in chemoattraction of proinflammatory [30] but obviously not anti-inflammatory cells in the course of atherogenesis.

Interestingly, in our study DCs and different subtypes of proinflammatory T cells positively correlated with the progression of atherosclerosis. Analyzing the frequency of DCs (CD83+, CD209+) in comparison with Tregs, a significant inverse correlation was observed. This is in agreement with the results of Dietel et al. [29] who also showed an inverse correlation. Assuming an anti-inflammatory function of Tregs [31], these observations of a concordant increase with proinflammatory cells and an inverse correlation with Tregs suggest DCs to function as an inductor of proinflammatory T cells and suppressor of Tregs in atherosclerosis.

Analyzing the distribution of Tregs in different plaque regions during plaque progression, we were able to show that Tregs are mainly present in the lipid core. Tregs present in unstable lesions seem to migrate into the lipid core and not to the same extent into the plaque shoulder regions where anti-inflammation would presumably be more important because these regions are the rupture-prone plaque regions.

Regarding the clinical data of the patients of the present study, one might notice that the percentage of male individuals is higher in the stable plaques group. Anyway, analyzing the cell numbers for men compared to women, there are no significant differences. There are no significant differences regarding cell numbers in carotid compared to femoral plaques. Interestingly, there is no difference in frequency of the examined immune cells considering cardiovascular risk factors or ischemic symptoms [13]. The reason for this difference might be the use of not only carotid but also femoral plaques for immunostaining in this study. Patients with peripheral arterial disease mainly suffer from chronic pain, but symptoms of acute vascular occlusion are rarely found.

Interestingly, there is an association of mDCs and Tregs with statin medication. Plaques of statin-treated patients showed a significantly lower number of DCs and a significantly higher number of Tregs. Even though not all studies demonstrated an anti-inflammatory effect of statins [32], our observations are in agreement with the majority of recent publications suggesting a plaque stabilizing effect of statin treatment [13, 33]. Regarding the different substances of statins, the dose-dependent effect of statin medication, or the effect of the duration of the statin treatment, we were not able to detect significant differences in the current study. This might be due to the size of the number of included patients.

In conclusion, the present study is one of the first showing a significantly lower number of Tregs in unstable atherosclerotic lesions compared to stable ones. This reduction in anti-inflammatory cells during atherogenesis might be an important reason for plaque destabilization. The increasing number of mDCs in the course of plaque progression, for which we were able to show a proinflammatory effect in the present study, might be the reason for a decrease in Tregs. This observation raises new questions about the interaction of DCs and Tregs which should be the point of interest in further studies.

## Figures and Tables

**Table 1:** Clinical data: cardiovascular risk factors and medications of the study groups.

	Group A	Group B	*P* value
	(*n* = 14, stable plaques)	(*n* = 15, unstable plaques)	
	(cells/0.1 mm^2^)	(cells/0.1 mm^2^)	
Age (years)	69.5 (57.8–78)	75 (72–77)	n.s.
BMI (kg/m^2^)	28.1 (25.3–29.7)	26.4 (24.5–29.1)	n.s.
Gender, male (%)	57	87	n.s.
Ischemic symptoms (%)	64	67	n.s.
Preoperative imaging:			
Degree of stenosis	80% (70–95%)	90% (80–95%)	n.s.
Blood parameters:			
Leukocytes (Gpt/L)	9.5 (6.7–14.4)	8.1 (7–10.1)	n.s.
CRP (mg/L)	9.3 (1–35)	2.1 (1–32)	n.s.
Creatinine (*μ*mol/L)	86 (66–157)	74 (60–133)	n.s.
Morbidities:			
Hypertension (%)	100	93	n.s.
Hyperlipidemia (%)	100	73	n.s.
Obesity (%) (BMI > 30 kg/m^2^)	14	20	n.s.
CHD (%)	21	53	n.s.
CKD (%) (GFR < 50 mL/min)	29	33	n.s.
Diabetes (%)	36	47	n.s.
Medication:			
ASA (%)	71	60	n.s.
Beta blocker (%)	43	73	n.s.
ACE Inhibitor (%)	43	60	n.s.
Statins (%)	92	60	n.s.

Group A: patients with stable, fibrous atherosclerotic lesions, group B: patients with unstable, lipid-rich atherosclerotic plaques. Values are presented as median and 25%–75% confidence interval or percentage.

^*^ACE: angiotensin-converting enzyme; ASA: acetyl-salicylic acid; BMI: body mass index; CHD: coronary heart disease; CKD: chronic kidney disease; CRP: C-reactive protein; GFR: glomerular filtration rate; LDL: low-density lipoprotein.

**Table 2:** Histological criteria for the determination of atherosclerotic plaques as “stable” or “unstable.”

	Stable plaque (SP)		Unstable plaque (UP)		*P* value
	Median	25–75% CI	Median	25–75% CI	
Fibrous cap (in *μ*m)	**267**	181–440	**96**	0–223	*0.02 *
LCR	**0.21**	0.08–0.28	**0.64**	0.54–0.76	*<0.001 *
Neovessels (/0.2 mm^2^)	**0**	0–0.3	**3**	3–5	*<0.001 *

^*^LCR: lipid core ratio; LCR: lipid core area/plaque area × 100.

**Table 3:** Antibodies used for immunohistochemical analyses.

Antibodies	Source	Dilution	Specificity
CD86	Milteyi Biotec	1 : 25	APCs
CD68	Dako	1 : 50	Macrophages
S-100	Dako	1 : 500	Glial Cells, ependyma, Schwann cells, mDCs
Fascin	Dako	1 : 100	mDCs
CD83	Serotec	1 : 40	Mature mDCs
CD209	BD Pharmingen	1 : 100	Immature mDCs
CD304	Miltenyi Biotec	1 : 100	pDCs
CD123	Serotec	1 : 100	pDCs
HLA-DR	Dako	1 : 25	APCs
CD3	Miltenyi Biotec	1 : 100	T cells
CD4	Dako	1 : 50	T helper cells
CD8	Dako	1 : 200	Cytotoxic T cells
CD161	Serotec	1 : 20	Natural killer cells
CD25	Invitrogen	1 : 20	Activated B and T cells, regulatory T cells
CD69	Ab cam	1 : 100	Activated B and T cells, regulatory T cells (early activation marker), macrophages, and platelets
FoxP3	BioLegend	1 : 20	Regulatory T cells
CD34	Dako	1 : 50	Endothelial cells
CCR4	BioLegend	1 : 50	Chemokine receptor of DC, T cells, and B cells
CCR-6	R&D	1 : 80	Chemokine receptor of immature DCs

^*^APC: antigen presenting cell; mDCs: monocytoid dendritic cells; pDCs: plasmacytoid dendritic cells.

**Table 4:** Frequency of pro- and anti-inflammatory cells in unstable and stable plaques.

IHC marker	Control vessel (CV)		Stable plaque (SP)			Unstable plaque (UP)			
	(cells/0.1 mm^2^)		(cells/0.1 mm^2^)		CV versus SP	(cells/0.1 mm^2^)		CV versus UP	SP versus UP
	Median	25–75% CI	Median	25–75% CI	*P* value	Median	25–75% CI	*P* value	*P* value
APC/DC									
CD68	**13.8**	5.0–19.8	**43.9**	27.0–74.4	*<0.001 *	**58.3**	25.2–98.2	*<0.01 *	n.s.
Fascin	**35.0**	24.1–46.9	**42.2**	20.0–55.8	n.s.	**67.2**	40.0–96.2	*0.01 *	*0.03 *
S100	**57.3**	6.4–108.8	**53.5**	25.3–73.4	n.s.	**87.0**	48.7–95.3	n.s.	*0.01 *
CD83	**9.3**	0.0–36.1	**14.9**	5.7–49.5	n.s.	**87.2**	31.5–102.3	*0.004 *	*0.003 *
CD209	**14.3**	6.0–31.8	**17.5**	9.2–23.9	n.s.	**43.5**	23.7–70.6	*0.01 *	*0.004 *
CD123	**3.3**	0.1–16.1	**2.4**	0.8–7.9	n.s.	**4.0**	0.5–6.8	n.s.	n.s.
CD304	**13.3**	0.0–38.5	**2.5**	0.5–14.1	n.s.	**4.0**	2.7–14.5	n.s.	n.s.
Functional APC marker									
HLA-DR	**32.0**	15.1–72.0	**49.1**	34.9–78.6	n.s.	**77.0**	48.4–119.6	*0.007 *	*0.03 *
T cells									
CD3	**12.3**	4.5–31.4	**24.3**	7.6–37.8	n.s.	**55.8**	44.5–75.3	*0.002 *	*<0.001 *
CD4	**9.0**	0.8–19.0	**5.5**	2.7–7.8	n.s.	**18.8**	6.5–29.0	n.s.	*0.008 *
CD8	**3.5**	0.5–9.5	**5.3**	2.9–10.3	n.s.	**18.0**	10.3–22.2	*0.001 *	*0.001 *
CD161	**4.0**	0.0–28.0	**16.8**	6.2–22.2	n.s.	**25.3**	14.1–37.2	*0.02 *	*0.03 *
Activated T cells									
CD25	**11.5**	0.5–23.0	**4.0**	1.2–13.3	n.s.	**26.3**	11.3–36.0	n.s.	*0.004 *
CD69	**1.3**	0.75–2.0	**2.3**	0.17–3.17	n.s.	**3.8**	2.5–9.5	n.s.	*0.04 *
Tregs									
FoxP3	**7.3**	3.8–43.8	**31.9**	16.3–49.8	*0.02 *	**9.0**	2.7–24.6	n.s.	*0.002 *
Chemokine Receptors									
CCR6	**38.0**	11.5–50.0	**36.3**	16.5–57.0	n.s.	**75.0**	48.5–92.0	*0.004 *	*0.007 *
CCR4	**32.5**	24.0–43.5	**43.0**	13.2–61.0	n.s.	**89.3**	54.0–102.5	*<0.001 *	*0.003 *

Values are presented as median and 25%–75% confidence interval or percentage.

^*^APC: antigen presenting cell; CCR: chemokine receptor; Tregs: regulatory T cells; CV: control vessel; SP: stable plaque; UP: unstable plaque.

**Table 5:** Correlation analyses of inflammatory cells, activation markers, and chemokine receptors.

Correlation between		Correlation coefficient	*P* value
Anti-inflamm. T cells	Proinflamm. cells		
FoxP3	CD83	−0.39	0.04
FoxP3	CD209	−0.43	0.02
FoxP3	CD4	−0.4	0.03
Proinflamm. T cells	DCs		
CD8	Fascin	0.55	0.002
CD4	Fascin	0.66	<0.001
CD3	Fascin	0.41	0.03
CD161	Fascin	0.61	<0.001
CD8	CD209	0.67	<0.001
CD4	S100	0.54	0.003
CD3	CD209	0.49	0.008
CD161	CD83	0.56	0.002
CD161	CD209	0.42	0.02
CD161	S100	0.44	0.02
T cell activation	DC activation		
CD25	HLA-DR	0.67	<0.001
Chemokine receptor	Proinflammatory cells		
CCR4	HLA-DR	0.46	0.01
CCR4	CD3	0.56	0.002
CCR4	S100	0.45	0.02
CCR4	CD83	0.43	0.02
CCR4	CD209	0.39	0.04
CCR4	CD4	0.45	0.02
CCR4	CD25	0.48	0.01
CCR4	CD3	0.56	0.002
Chemokine receptor	Anti-inflamm. T cell		
CCR4	FoxP3	−0.38	0.04

## Abbreviations

APC: Antigen-presenting cell
CCR: Chemokine receptor
DC: Dendritic cells
ECM: Extracellular matrix
FoxP3: Forkhead box protein 3
IFN: Interferon
LDL: Low-density lipoprotein
oxLDL: Oxidized low-density lipoprotein
Tregs: Regulatory T-cells.
